# Rosie, a Health Education Question-and-Answer Chatbot for New Mothers: Randomized Pilot Study

**DOI:** 10.2196/51361

**Published:** 2024-01-12

**Authors:** Quynh C Nguyen, Elizabeth M Aparicio, Michelle Jasczynski, Amara Channell Doig, Xiaohe Yue, Heran Mane, Neha Srikanth, Francia Ximena Marin Gutierrez, Nataly Delcid, Xin He, Jordan Boyd-Graber

**Affiliations:** 1 Department of Epidemiology and Biostatistics University of Maryland School of Public Health College Park, MD United States; 2 Department of Behavioral and Community Health University of Maryland School of Public Health College Park, MD United States; 3 Department of Computer Science, University of Maryland Institute for Advanced Computer Studies University of Maryland College Park, MD United States

**Keywords:** chatbot, health information, maternal and child health, health disparities, health equity, health informatics, preventive health care, postpartum care, patient education, newborn care, prenatal care, mobile phone

## Abstract

**Background:**

Stark disparities exist in maternal and child outcomes and there is a need to provide timely and accurate health information.

**Objective:**

In this pilot study, we assessed the feasibility and acceptability of a health chatbot for new mothers of color.

**Methods:**

Rosie, a question-and-answer chatbot, was developed as a mobile app and is available to answer questions about pregnancy, parenting, and child development. From January 9, 2023, to February 9, 2023, participants were recruited using social media posts and through engagement with community organizations. Inclusion criteria included being aged ≥14 years, being a woman of color, and either being currently pregnant or having given birth within the past 6 months. Participants were randomly assigned to the Rosie treatment group (15/29, 52% received the Rosie app) or control group (14/29, 48% received a children’s book each month) for 3 months. Those assigned to the treatment group could ask Rosie questions and receive an immediate response generated from Rosie’s knowledgebase. Upon detection of a possible health emergency, Rosie sends emergency resources and relevant hotline information. In addition, a study staff member, who is a clinical social worker, reaches out to the participant within 24 hours to follow up. Preintervention and postintervention tests were completed to qualitatively and quantitatively evaluate Rosie and describe changes across key health outcomes, including postpartum depression and the frequency of emergency room visits. These measurements were used to inform the clinical trial’s sample size calculations.

**Results:**

Of 41 individuals who were screened and eligible, 31 (76%) enrolled and 29 (71%) were retained in the study. More than 87% (13/15) of Rosie treatment group members reported using Rosie daily (5/15, 33%) or weekly (8/15, 53%) across the 3-month study period. Most users reported that Rosie was easy to use (14/15, 93%) and provided responses quickly (13/15, 87%). The remaining issues identified included crashing of the app (8/15, 53%), and users were not satisfied with some of Rosie’s answers (12/15, 80%). Mothers in both the Rosie treatment group and control group experienced a decline in depression scores from pretest to posttest periods, but the decline was statistically significant only among treatment group mothers (*P*=.008). In addition, a low proportion of treatment group infants had emergency room visits (1/11, 9%) compared with control group members (3/13, 23%). Nonetheless, no between-group differences reached statistical significance at *P*<.05.

**Conclusions:**

Rosie was found to be an acceptable, feasible, and appropriate intervention for ethnic and racial minority pregnant women and mothers of infants owing to the chatbot’s ability to provide a personalized, flexible tool to increase the timeliness and accessibility of high-quality health information to individuals during a period of elevated health risks for the mother and child.

**Trial Registration:**

ClinicalTrials.gov NCT06053515; https://clinicaltrials.gov/study/NCT06053515

## Introduction

### Background

Maternal morbidity and mortality have remained persistent problems in the United States and disproportionately affect women and birthing people from racial and ethnic minoritized backgrounds owing to embedded racism and bias across the medical and public health systems [1-3]. More concerningly, >80% of maternal deaths in 2019 were designated as preventable by the Centers for Disease Control and Prevention’s (CDC’s) maternal mortality review committees [4]. In particular, the perinatal period is associated with high risk of depression and anxiety among mothers and is a leading cause of maternal mortality in the United States [1,4,5]. Other leading causes include hemorrhage, cardiovascular and coronary conditions, and substance use disorders [4,5].

Timely and reliable health information may help to reduce the adverse outcomes during pregnancy and in the postpartum period [6]. Children of single parents, with low household income, of a minority group, or whose parents perceived them as being more susceptible are seen more frequently in the emergency department [7-10]. Health education interventions have been shown to reduce emergency department use among infant caregivers [11]. Seeking health information on websites is common among soon-to-be and new parents; however, the quality of information and sources found on the web about pregnancy, birth, parenting, and maternal health were rated by pregnant women and new parents as having varying quality, or the information found was not sufficiently specific to fully answer questions [12,13]. Currently, some popular programs for these vulnerable populations involve resource-intensive home visits, which face challenges in scaling to assist more mothers owing to staff and cost constraints, or nonpersonalized SMS text messages that may not directly address an individual’s questions [14-18].

Recognizing that facilitating maternal and child health equity in the United States will require intervention at all levels of the socioecological model to address the deficits in medical and public health research and practice, our research team selected an innovative approach. This approach consisted of developing a maternal and child health information chatbot that would be iteratively improved through a multiyear, community-engaged research process. The chatbot addresses some limitations of previous strategies by providing personalized health information based on the users’ needs, is readily available at any time, and can include participants nationwide. Rosie offers timely health information from verifiable websites such as children’s hospitals or the CDC to help parents navigate infant care and find clinically correct information to tackle health issues as they arise. In addition, Rosie offers reminders about preventive care visits for infants (eg, well-baby visits) that can also encourage greater continuity of care, which have been shown to reduce emergency department visits for infants [19].

The research team developed the chatbot, named Rosie, to be able to respond to user questions about parenting, pregnancy, and infant development with vetted, trustworthy web-based sources. Question-answering (QA) chatbots such as Rosie, unlike informational sites and frequently asked questions (FAQs) pages, support their users with personalized responses based on the user’s input. They provide users with the unique opportunity to enter their questions in their own words and receive responses to their questions. We built the corpus or index of maternal and child health information using the information derived from expert sources such as federal agencies (eg, the National Institute for Child and Human Development), hospitals (eg, Mayo Clinic), and professional medical organizations (eg, the American Academy of Pediatrics). These sources provided vital information regarding topics such as pregnancy, parenting, infant development, maternal health, and postpartum care.

Chatbots developed to support maternal mental health and parenting have been shown to be a promising intervention needing further evaluation [20-22]. A mixed methods review of literature led by Chua et al [20] suggests that maternal and child health information chatbots have high acceptance among pregnant women and new parents; however, the reviewed papers noted that both development teams and test users expressed preferences for refining the language used in the responses to be more humanlike and for the chatbots to be familiarized with informal, descriptive language to be more adept at generating answers for users who may be describing symptoms or may not know the medical term for the topic of interest. Recognizing these recommendations from the literature, our research team used a multimethod approach to receive substantive, high-quality feedback from participants when launching our pilot evaluation of our maternal and child health information chatbot, Rosie.

### Study Objectives

We conducted an experimental pilot study to examine the feasibility, acceptability, and appropriateness of Rosie for our target audience. The pilot study also allowed us to test all the software and equipment, study protocols, and staff coordination to enable remedies before scaling to a full randomized controlled trial. Pilot data were also used to enhance the accuracy of Rosie’s responses by refining the existing models and fine-tuning the mechanisms and heuristics. The focus was on determining access to the resources and the capability of implementing the components and activities of the intervention as planned. Challenges in the provision of any component or the performance of any activity of the intervention were identified and potential solutions were determined. A sample size of 30 was chosen to be within the typical sample size range of a phase 1 clinical trial, according to the National Institutes of Health definitions, to conduct the study protocols and elicit participant feedback about the Rosie app with sufficient representation from our target group. Preintervention and postintervention tests were completed to qualitatively and quantitatively evaluate Rosie and describe any changes across key health outcomes including postpartum depression and frequency of emergency room visits to inform the full trial’s sample size calculations. Analyses of these data allowed us to present the preliminary findings, which should be interpreted as preliminary evidence, given that the pilot study was not powered to assess treatment-control differences, and this was not the main objective of the pilot study.

## Methods

### Development and Functionality of Rosie, the Chatbot

Rosie was customized to meet the needs of the target audience through continuous community feedback. Over the course of 3 years, our research team conducted community listening sessions, >20 community demonstrations of Rosie [23], and focus groups with pregnant women and new mothers of color. With this feedback, we customized Rosie to respond to health topics that mothers requested such as feeding tips, sleep advice, and information about rashes and fevers. To the Rosie app, we also added a set of the most popular questions that were asked by mothers as an FAQs page and provided a list of additional resources (eg, Supplemental Nutrition Assistance Program) benefits and emergency hotlines). Moreover, we added the requested video library that features things such as how to swaddle a baby, change a diaper, or perform cardiopulmonary resuscitation.

To build Rosie’s robust knowledge base, we collected, scraped, and extracted text from 60 sources, including websites of government agencies, hospitals, and professional medical organizations. A corpus of documents about maternal and infant health was built by scraping text from these vetted web domains using Trafilatura, a Python package and a web document processing tool called Scrapy that extracts text from HTML source code [24]. Each web document was then parsed into approximately 73,000 passages by applying a set of heuristics that retain sentence context. These passages were edited as necessary to serve as answers to the mothers’ questions and were used in a question generation model, probably asked questions (PAQs), to produce likely questions from users. The generated questions and their source passages were reviewed by annotators, who either edited both the question and the passage as necessary or discarded the pairs that were unhelpful, inaccurate, or incomprehensible. The answers augmented the existing knowledge base.

In addition, the research team supplemented the knowledge base by manually writing 350 question-and-answer pairs based on feedback from focus groups and community events with pregnant women and new mothers of color, who asked Rosie questions and identified topics of particular interest (eg, rashes and infant sleep). Only verified sources of health information were used in Rosie’s knowledge base. Sources with sponsored or commercial content were excluded.

Rosie’s underlying QA system uses an unsupervised, dense passage retrieval model. When users ask questions to Rosie, the retrieval model finds relevant content from the knowledge base that best answers the questions. Rosie also provides a source link in her responses, which can direct users to the websites from where the answer was extracted.

To better communicate with the users and understand their needs, we implemented an intent classification model using Rasa, a conversational artificial intelligence software. This classification model is used to categorize users’ text and respond accordingly. For example, it can identify greetings, thank you messages, and requests for information. It is also able to detect potential mental and physical health emergencies and send alerts via Slack, an instant messaging program, to our team members. Upon detection of a possible health emergency, Rosie sends emergency resources and relevant hotline information. In addition, a study staff member, who is a clinical social worker, reaches out to the participant within 24 hours to follow-up.

**Figure 1:**
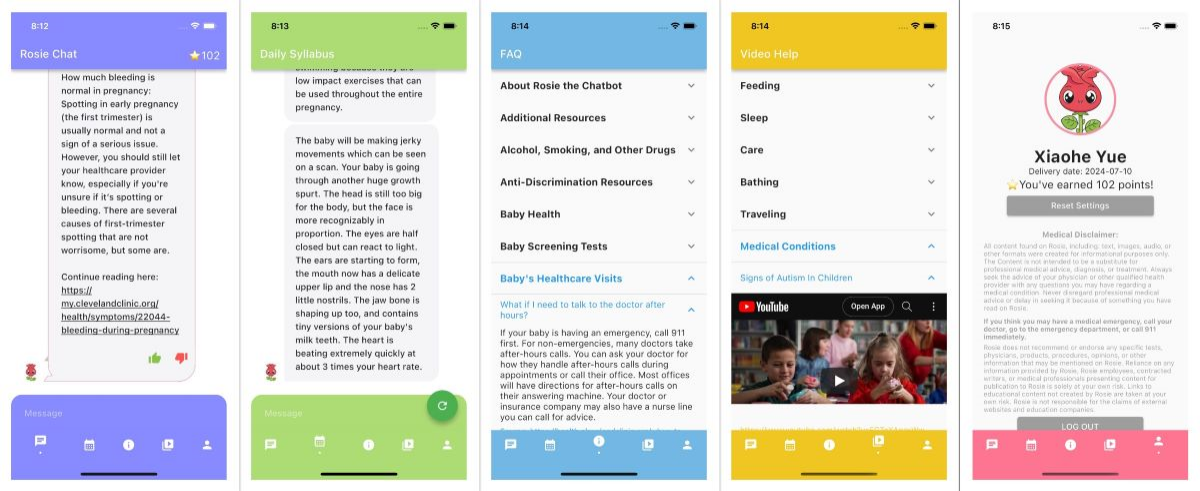
Rosie mobile app interface.

Rosie was built using Flutter, an open-source user interface software platform by Google, and designed to be compatible with both iPhone and Android devices. The Rosie mobile app has a log-in page with Google authentication, a chat window page that allows users to ask Rosie questions and rate the answers, an FAQs and resources page, and a medical disclaimer page reminding users that Rosie is an informational tool that does not replace professional medical advice and care (Figure 1).

After downloading and logging into the Rosie app, users are asked to enter their estimated due date or their infant’s date of birth. With the users’ permission, Rosie sends push notifications with daily health tips that are generated based on the due date or birth date, thus allowing for personalized advice based on the week-by-week progression of the user’s pregnancy or infant’s health. The past 7 days’ tips are also saved on an app page for users to refer to if needed. The app development team also created an app monitoring system that can detect server-related issues or app interruptions and notify team members via Slack for prompt troubleshooting and resolution. All conversations between users and Rosie were securely stored in Firebase Database.

### Recruitment and Enrollment

This was a prospective randomized controlled pilot study involving a mobile app intervention, Rosie the chatbot. To clarify the methods, we followed the CONSORT (Consolidated Standards of Reporting Trials) checklist (Multimedia Appendix 1). From January 9, 2023, to February 9, 2023, participants were enrolled on a rolling basis until the target sample size was met (N=30) for the 3-month randomized pilot study. Participants were recruited using social media posts, including targeted advertisements, and through partnerships with community-based organizations. In addition, our research assistants contacted mothers who had completed an interest form at a previous Rosie community event or focus group.

Interested potential participants completed a screening questionnaire to determine eligibility. Inclusion criteria included being aged ≥14 years, being a woman of color, and either being currently pregnant or having a baby aged ≤ 6 months. Research assistants contacted each of the 248 potential participants to complete a brief video call to assess eligibility, explain study details, and obtain informed consent for participation. We identified fraudulent interest forms through video calls and review of interest survey meta-data, including IP addresses, to filter out potential participants who were falsely claiming to meet the inclusion criteria or were residing outside the United States.

**Figure 2:**
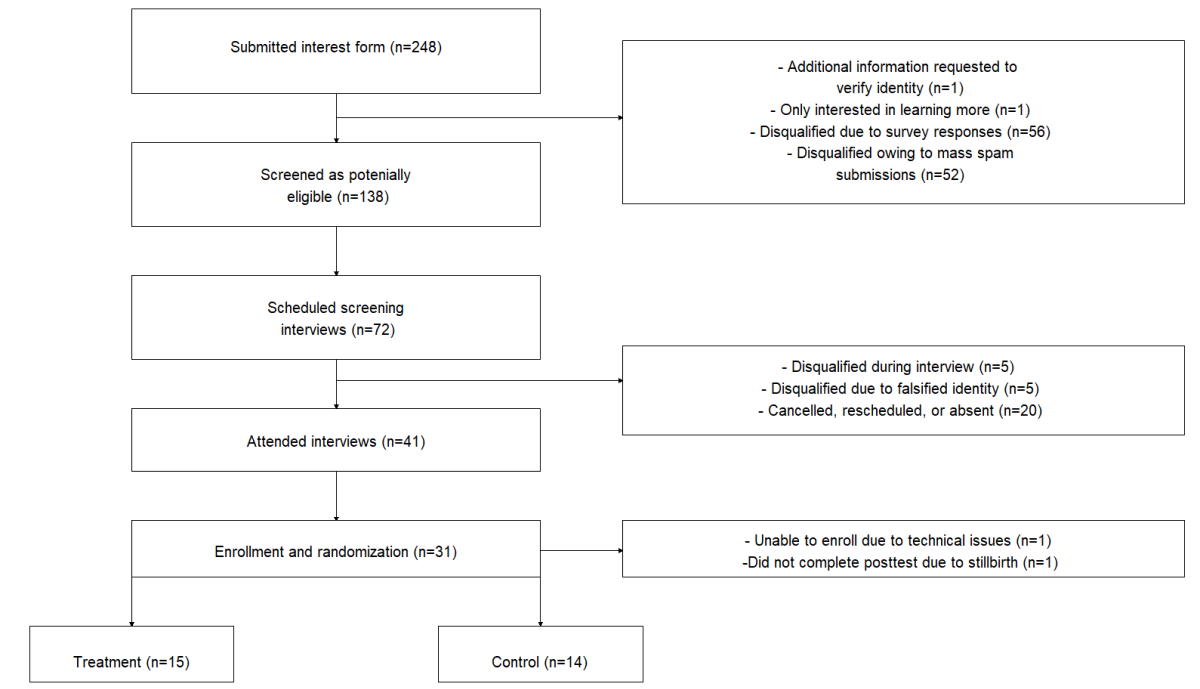
CONSORT (Consolidated Standards of Reporting Trials) diagram of participant enrollment.

Eligible participants were randomized into the control group (a monthly children’s book club) or the treatment group (Rosie, the chatbot) using a web-generated table with 15 slots for each study arm, for a total of 30 enrolled participants. After a participant assigned to the Rosie treatment group was unable to fully enroll owing to technical issues, we recruited an additional participant as a replacement. In addition, a mother in the control group experienced a stillbirth during the pilot study and did not complete the postintervention test. Thus, the final analytic sample was 52% (15/29) Rosie treatment group members and 48% (14/29) control group members (Figure 2). Among the 29 participants who were successfully recruited for the pilot study, 3 (10%) were recruited from partner organizations, 6 (21%) were recruited based on our interest forms at past Rosie events, and the remainder (n=20, 69%) were recruited using social media advertisements.

The control group participants were mailed a children’s board book once a month. The control group was modeled after similar programs across the United States that provide free monthly books to children [25]. Books were selected based on feedback from focus groups and community demonstrations with new parents or pregnant women of color, who expressed a desire to have books featuring diverse families.

At the initial enrollment meeting, our research team provided the Rosie treatment group participants instructions about how to install the Rosie app on their smartphones and provided a walkthrough of how to use the app and how to provide feedback about Rosie’s responses to their questions. We emailed each participant a weekly user engagement summary and sent reminder SMS text messages to encourage the use of the app.

### Data Collection, Outcome Measures, and Analysis

We assigned each participant an identification number to link pretest and posttest data and to track progress through the pilot study using clinical trial management software. Enrolled participants completed a pretest and posttest Qualtrics survey. Pretest surveys included questions about demographics (maternal age, race and ethnicity, education, household size, and health insurance), whether they were pregnant or parenting a young infant, and their due date or their baby’s birth date (as applicable). Pretest surveys also included the Patient Health Questionnaire–9 (PHQ-9) depression scale [26] and assessed the frequency of emergency room visits for infants.

Posttest surveys administered at the 3-month follow-up assessed pregnancy outcomes (birth weight and gestational age), emergency room visits for infants, PHQ-9 depression scale, and group-specific questions. Rosie treatment group members were asked how often they used Rosie and whether they experienced any of the following issues while using the app (eg, “application crashed,” “took too long to get a response,” “was difficult to use,” and “was not satisfied with the answer[s] to my question[s]”). In contrast, the control group members were asked to rate how much they agreed with the following statements on a 5-point Likert Scale ranging from 5 (strongly agree) to 1 (strongly disagree): the books I received were of good quality, the content of the books I received is a good match for my baby’s needs, the books were helpful to me during my pregnancy or parenting my infant, the books were enjoyable to me during my pregnancy or parenting my infant, participating with the book club was easy for me, and I would recommend the book club to other parents.

Both groups were also asked open-ended questions to obtain qualitative feedback. Rosie treatment group members were asked the following open-ended questions: (1) Besides answering your questions, what other features would you like to see on an application like Rosie? (2) Do you have any concerns about using Rosie? If so, please tell us about them; and (3) Do you have any additional feedback to help us build the best Rosie app possible? In an open-ended question, the control group members were asked to provide any additional feedback or ideas about their experience with the book club.

Descriptive statistics of the study sample were calculated based on group assignment. Pretest and 3-month posttest values were examined for postpartum depression and emergency room visits for infants. To examine the statistical significance of between-group differences at baseline, Fisher exact tests were used for categorical variables and Wilcoxon rank sum tests were used for continuous variables. For the within-group pretest and posttest comparisons, paired 2-tailed *t* tests and McNemar tests were applied. Furthermore, 2-sample 2-tailed *t* tests were used to compare the between-group differences in the pretest to posttest changes. Qualitative feedback was organized and presented separately for the Rosie treatment group and book club control group members.

### Ethical Considerations

The consent form was read aloud by research assistants to each participant and verbal informed consent was obtained. Written consent was obtained through the completion of an web-based form, and a copy of the consent form was sent via email to each enrolled participant. Participants were also encouraged to use the app and were told that if they were continuously enrolled for 3 months and actively engaged with the app by asking questions to the chatbot at least once a week, they would receive a gift card worth US $50. In addition, participants were told that if, at the end of the 3-month pilot study, they were among the top 20% of active users, defined by the number of unique questions sent to Rosie, they would also receive a tablet preloaded with children’s books. Participants were also given a gift card worth US $15, disbursed through a participant incentive distribution platform, Tango, upon completing the pretest and posttest Qualtrics surveys. The study was reviewed and approved (institutional review board study ID: 1556200) by the institutional review board of the University of Maryland, College Park, based on procedures for studies involving human participants.

## Results

### Overview

Baseline key demographic characteristics were not statistically significantly different between the Rosie treatment and control groups (Table 1). The mean age of mothers for both groups was 31.7 (SD 4.7) years. Approximately half of the mothers (9/15, 60%) were pregnant, and the other half (6/15, 40%) took care of young infants. Among those with infants, the mean age of infants was 4 months among Rosie treatment group members and 4.6 months among control group members. Most participants (9/15, 60%) were African American or Black, with the remainder being Asian, Hispanic or Latina, or multiracial (Table 1).

**Table 1:** Total enrollment demographics^a^.

Participant characteristics		Rosie (treatment group; n=15)	Book club (control group; n=14)	*P* value	
Age (years), mean (IQR)		31.7 (30-35)	31.6 (26-35)	.83	
**Race and ethnicity, n (%)**					.14
	Asian	1 (7)	4 (29)		
	African American or Black	9 (60)	10 (71)		
	Hispanic or Latino	5 (33)	2 (14)		
	Multiracial	2 (13)	1 (7)		
Currently pregnant, n (%)		9 (60)	6 (43)	.36	
Parenting infant, n (%)		6 (40)	8 (57)	.19	
Currently pregnant and parenting infant, n (%)		0 (0)	1 (7)	.29	
Infant age, months (Q1-Q3)		3.7 (2-5)	4.8 (4-6)	.15	
**Education, n (%)**					.37
	High school	2 (13)	2 (14)		
	Associate degree	1 (7)	2 (14)		
	Bachelor degree	3 (20)	5 (36)		
	Master degree	3 (20)	4 (29)		
	Professional degree	6 (40)	1 (7)		
Average family size, mean (range)		2.6 (2-3)	2.9 (2-3)	.49	

^a^To examine the statistical significance of between-group differences at baseline, Fisher exact tests were used for categorical variables and Wilcoxon rank sum tests were used for continuous variables.

### Acceptability of Rosie

More than 87% (13/15) of Rosie treatment group members reported using Rosie daily (5/15, 33%) or weekly (8/15, 53%) across the 3-month study period. Most users reported that Rosie was easy to use (14/15, 93%), and that they received a response from Rosie quickly (13/15, 87%). The remaining issues identified included crashing of the app during attempted use (8/15, 53%), and they were not satisfied with some of Rosie’s answers (12/15, 80%; Table 2).

**Table 2:** Acceptability statistics for Rosie treatment group (n=15).

Questions and response options			Participants, n (%)
**How often did you use Rosie?**			
	Monthly or less	1 (7)	
	Multiple times a day	1 (7)	
	Once daily	5 (33)	
	Weekly	8 (53)	
**Did you experience any of the following issues while using the app?**			
	Application crashed	8 (53)	
	It took too long to get a response	1 (7)	
	It was difficult to use	2 (13)	
	I was not satisfied with the answer(s) to my question(s)	12 (80)	

### Health Results: Quantitative

Pilot results suggested better health outcomes for the Rosie treatment group compared with the control group; however, between-group differences did not reach statistical significance. The estimated change in Rosie participants’ PHQ-9 mean depression scores from baseline to posttest period was −3.66 (SD 4.55) among Rosie treatment group participants compared with −2.77 (SD 4.92) among control group members (Table 3). This decline in depression scores between pretest and posttest period was only statistically significant for the Rosie treatment group (*P*=.008) and not the control group (*P*=.07). None of the participants from either group reported any emergency room visits for infants at baseline, but this percentage increased to 23% (3/13) for the control group members versus 9% (1/11) for the Rosie treatment group members (Table 3). Notably, 10 (67%) out of 15 mothers who were pregnant at baseline gave birth by the 3-month posttest period, and it could be a possible reason why emergency room visits for infants increased during the posttest period for both groups.

**Table 3:** Health and health behavior outcomes^a^.

			Rosie (treatment group)		Book club (control group)		*P* value	
**Maternal depression scale^b^**								N/A^c^
	Pretest period, mean (SD)	5.33 (4.43)		5.31 (3.33)		N/A		
	Posttest period, mean (SD)	1.67 (2.64)		2.54 (2.96)		N/A		
	Post-pre change, mean (SD)	–3.66 (4.55)		–2.77 (4.92)		.62		
	Values, n (%)	15 (100)		13 (100)		N/A		
**Any emergency visit for infants**								N/A
	Pretest period, n (%)	0 (0)		0 (0)		N/A		
	Posttest period, n (%)	1 (9)		3 (23)		N/A		
	Pre-post change	+9.09		+23.08		.60		
	Values, n (%)	11		13		N/A		

^a^For the within-group pretest and posttest comparisons, paired 2-tailed *t* tests and McNemar tests were applied. Paired 2-tailed *t* tests comparing pretest and posttest Patient Health Questionnaire depression scores were statistically different for the treatment group (*P*=.008). No other within-group comparisons were statistically significant at *P*<.05. Moreover, 2-sample 2-tailed *t* tests were used to compare the between-group differences in the pretest to posttest changes. *P* values assess pre- to postperiod changes for treatment versus control groups.

^b^The sample size for emergency room visits was smaller because this outcome was assessed among mothers with infants (excludes currently pregnant mothers during the posttest period).

^c^N/A: not applicable.

### Rosie Results: Qualitative Feedback

The Rosie participants provided considerable qualitative feedback about their experiences (Textbox 1). Participants expressed that they liked having a personal library to ask all their pregnancy and parenting questions, but improvements were needed in both the user experience and the content of responses. Participants commented that the quality of Rosie’s responses to pregnancy-related questions seemed to be low in accuracy compared with questions about infant caretaking. Participants noted that they observed improvements in the app’s functionality as the trial progressed and that the addition of an FAQs library and daily tips about baby’s development were helpful.

**Qualitative feedback about Rosie.**

**Domain and feedback**

Strengths

- “I really enjoyed having a personal library to ask all the questions. Especially because after losing my baby, I didn’t receive a ton of targeted ads since my questions were limited to this app.”
- “I liked it when the daily tips were added to the app. Before that, they were only notifications that I couldn’t go back to and often couldn’t fully read. I also liked when the previous day’s answers became available for viewing, so I didn’t have to screenshot or save links.”
- “I like everything else.”

Points system

- “Additionally, the point system didn’t fit with the original study. We were asked to use Rosie a minimum of once a week with a preference for more frequent use. But then the points were awarded for daily use. It was discouraging to not earn points, but with a newborn at home it was a struggle to do anything other than feed him daily.”

Content and technical concerns

- “Perhaps some answers were not very accurate for the age of my baby, they were for younger or older kids.”
- “Answers were not specific enough, lots of glitches with the app.”
- “The answers to the questions were often inaccurate.”
- “It does not answer me well.”
- “Not efficient. All of the answers take you to the same website. Using the app was a waste of time.”
- “My pregnancy-related questions were often answered inaccurately. The baby questions were mildly better, but if the chatbot is intended for both than it needs more training related to pregnancy symptoms and side effects.”
- “I had some trouble with the push notifications as well. At first, they were accurate for the number of weeks along I was in my pregnancy. Then they started to speed up, telling me that I was as many as two weeks ahead of my baby’s gestational age [e.g., it said I was 38 weeks when I was really 36]. I went in and reset it, using the same due date that I started with, and it continued to be incorrect. The app itself didn’t seem to have the same problem, just the notifications.”
- “Due to the high number of inaccurate responses, I was not motivated to continue using the app. I tried to stick with it, but to be honest this chatbot and the accompanying app have a long way to go before they’re ready for implementation.”
- “Finally, last week I had some issues with the app where it was giving me answers in a mix of text and source code formatting. Everything looked like a hyperlink but the links themselves did not work. I received an update notification, updated the app, and the problem persisted. It did fix itself after a couple of days though.”
- “At times when typing a question at the moment of submitting the keyboard would stay open and would not allow the user to hit submit. I had to exit out the app completely and reopen it and it would work again.”
- “Sometimes I had urgent questions, it could’t be use because it was under maintenance often.”
- “Hard to update.”
- “Rosie crashed a few times [like over multiple days when I tried asking a question].”

Suggestions

- “Sometimes Rosie gave me some answers that were not related to what I wanted to know, I understand sometimes terms can apply to two different things but perhaps Rosie can ask Do you mean this (1) or this (2), and then one chooses what is closer to the question one is asking. It happened to me a couple of times but I don’t remember the specific question.”
- “Answers were not specific enough, lots of glitches with the app.”
- “More accuracy with the answers would be great!”
- “Just implement user feedback.”
- “It would be nice to see some statistics [e.g., x% of kids do x by whatever age].”
- “I was pretty unlikely to visit the website Rosie referred me to. I think I would be more likely to view info right on the screen [even a click box with additional text or pics etc].”
- “Random suicide hotline warning was a little bit abrupt and unexpected.”
- “Answers were unrelated sometimes.”
- “Maybe something more personal with the week we are on if its more geared for pregnancy.”
- “I would say maybe [a] different app for moms and another version for expecting moms only due to the fact that some symptom questions were meant for a child rather than me who is pregnant. Overall the app is a great idea and good help/support for all. Thanks for the opportunity.”
- “When I ask questions, it didn’t answer my answers so I suggest to add more keywords for more accurate answers.”
- “Live chat.”
- “Maybe a bilingual app?”
- “A way to keep a record of my baby’s weight and height.”
- “Citations, more images, voice feature.”
- “Chance to chat with other moms and create a community of peer to peer questions and answer library. I really wanted to talk to other moms who had experienced loss early in their pregnancy too.”
- “It should have a lot of tips for healthy living.”
- “Sometimes I would forget what question I already asked because the previous questions would disappear. It could be nice to have an archive of questions.”
- “An actual calendar.”

As a result of the feedback, the development team has added more source websites to Rosie’s knowledge bank and has expanded the FAQs section of the app to include topics such as descriptions of the full schedule of well-baby visits and immunizations in the first 2 years of life. As this pilot study was designed to be part of a broad iterative process, negative, neutral, and positive feedback are all integral parts of refining the app’s functionality and expanding its knowledge base.

### Book Club Results: Qualitative Feedback

Participants were extremely positive in their feedback about the book club, with participants rating the books as being of high quality and as a helpful tool for parenting (Textbox 2 and Table 4). All participants (14/14, 100%) agreed that they would recommend the book club to other new parents. Participants appreciated the “diversity and bilingual aspects of the books” and that their infants “really enjoyed the Global Babies book and loved to stare at the faces [presented in the books].” A participant offered a recommendation for an additional children’s book by an author whose works focus on social justice leaders in the United States to be offered in the book club.

**Qualitative feedback about the book club.**

**Domain and feedback**

Strengths

- “I so much love it.”
- “I loved the diversity and bilingual aspects of the books.”
- “Baby really enjoyed the global babies book and loved to stare at the faces.”

Suggestions

- “The book[s] were good, but I think there are more popular/exciting book options for babies, especially books featuring babies of color. I am thinking of all the books by Jabari Asim for example. The last book was great though and the overall idea for a book club is fantastic. I loved knowing that new books were coming each month.”
- “Oh, another thing: I got an automatic message from the book club quite frequently with the same message and it seemed redundant.”

**Table 4:** Acceptability statistics for the control group (n=13).

Item		Score, mean (SD)
**Please rate how much you agree with the following statements based your thoughts and experiences (1=strongly disagree; 5=strongly agree)**		
	The books I received were of good quality	4.54 (0.14)
	The content of the books I received is a good match for my baby’s needs	4.46 (0.18)
	The books were helpful to me during my pregnancy or parenting my infant	4.38 (0.29)
	The books were enjoyable to me during my pregnancy or parenting my infant	4.58 (0.19)
	Participating with the book club was easy for me	4.77 (0.12)
	I would recommend the book club to other parents	4.85 (0.10)

### Health Results: Qualitative

A Rosie treatment group participant and a control group participant experienced pregnancy loss (a miscarriage and a stillbirth) during the study. An unexpected finding from one of the participants, who was assigned to the treatment group, was that using Rosie for maternal and child health information helped in shielding them from some emotional distress after their loss, with our participant stating the following:

I really enjoyed having a personal library to ask all the questions. Especially because after losing my baby, I didn’t receive a ton of targeted ads since my questions were limited to this app.

Recognizing that most of the currently available maternal and child health apps track user interactions for advertisers and feature advertisements, Rosie’s development as a no-cost, advertisement-free app may have additional benefits for mothers who value personal data privacy.

## Discussion

### Principal Findings

The pilot study demonstrates that Rosie is a feasible, acceptable, and appropriate intervention for pregnant women and new mothers of color. Rosie’s software was able to function with a given set of users and was generally able to generate responses to most of the asked questions. Our study found overall reduction in the PHQ-9 depression scale scores from baseline to the 3-month follow-up across both groups. However, the Rosie treatment group experienced a relatively large reduction from baseline to posttest follow-up. The reduction in maternal depression among both groups may correspond to known trajectories in maternal depression, which identified that depressive symptoms peak around birth and decrease as the infant ages [27,28]. Researchers have found variability in the timing and duration of perinatal and antenatal depression, but some studies have found that women who had depression symptoms during the antenatal period were likely to have more intense symptoms during pregnancy than during the postpartum period and that perinatal depression symptoms decreased over time [29,30]. With an expanded time frame of 12 months planned for intervention delivery during the full randomized controlled trial, the research team will be more able to precisely track trends in depressive symptoms and identify what, if any, congruence exists in our participants and the current literature about maternal depression and other mental health symptoms.

Although our small sample size and study design limit our ability to identify the causal pathways between Rosie and changes in depressive symptoms, our findings indicate that an association may exist between the use of the Rosie app and low maternal depression owing to increased parental confidence about their own health and infant caretaking through increased access to accurate health information. Rosie’s ability to provide rapid, accurate response with high-quality sources may also reduce the cognitive burden that pregnant women and new parents described in previous studies that emphasized that sorting through information, making comparisons, and determining the quality of the source of information were significant stressors. In addition, the Rosie app may also reduce maternal depression because it can help provide support to mothers who may not otherwise have access to many health-related supports and resources.

The low rates of emergency room use for infants in the Rosie treatment group compared with the control group aligns with previous study hypotheses that health information provided by Rosie can decrease acute health care use. Nonetheless, this could have occurred through multiple channels including potentially greater use of preventive health care services and Rosie assisting with the identification of relevant health information or clinical guidelines to support infant care.

The qualitative feedback the Rosie participants provided aligns with the conclusions obtained by Chua et al [20] during their review of maternal health chatbots that the first evaluations of these interventions often yield a need for improvement in the language models to understand the variety of ways in which users may ask questions about their pregnancy and child and to provide more precise and accurate responses to these questions.

### New Rosie Features in Response to the Pilot Study

User experiences and feedback about the Rosie app has informed the further development of Rosie and continued precision of the QA model. For each of Rosie’s responses, users were able to click “thumbs up” or “thumbs down” to indicate their satisfaction or dissatisfaction with Rosie’s response to their question, and approximately 35% of questions received this additional level of feedback from users. The team analyzed this feedback and enhanced Rosie’s knowledge base by including topics that were not covered in previous iterations of the QA model and further refined the QA model based on the issues identified by the participants and the research team. We analyzed and discussed these interactions weekly with the goal of improving Rosie and initiating improvements in the user experience.

In addition, as a result of user feedback, we have expanded Rosie’s knowledge bank by >10 folds from 75,000 passages to >1.8 million passages extracted from 400,000 documents from verified health sources such as the CDC, National Institutes of Health, Mayo Clinic, and children’s hospitals. Rosie’s previous knowledge bank was restricted to only maternal and infant care questions, but Rosie users had requested information about topics such as managing chronic conditions, food safety and preparation, mental health, and self-care. The expanded corpus now enables mothers to ask any health-related question.

### Strengths

This pilot study adds to the existing literature about chatbots broadly and their application in the context of maternal and child health. The team’s findings specific to reduction in maternal depression will help address one of the CDC-identified preventable causes of maternal death. In addition, low emergency room visits for infants suggest potential improvements in infant care and avoidance of some health crises. Our qualitative findings concur with those of previous studies, showing that improved precision in responses is needed [13,20]. Overall, participants found the chatbot as a helpful tool, and this intervention is delivered in a way that is easily accessible and usable. They also believe that it is an appropriate and acceptable approach for women of color who are pregnant or parenting an infant to receive reliable information. The feedback from our participants is invaluable in the preparation for scaling to a full randomized controlled trial. The use of a multimethod approach that obtained both quantitative and qualitative feedback resulted in a broad understanding of participants’ experiences and needs and addressed some gaps recognized in previous trials of chatbots designed for improving health knowledge.

### Limitations

The recruitment of study participants was conducted primarily through web-based advertisements, potentially yielding a sample of participants who are overall more comfortable with using apps and their phones as their primary way of seeking health information than other women who are pregnant or parenting infants. Our sample, overall, was highly educated and the most (26/29, 90%) had health insurance, which may have reduced our ability to detect the experiences and health information needs of mothers without the same level of education or identify the needs of mothers whose lack of insurance may be associated with more variability in the use of emergency rooms. The small sample size of this pilot study resulted in low statistical power. Several health outcomes were found to be different in the posttest period when making between-group comparisons of the Rosie treatment and control groups, but differences did not reach statistical significance. In addition, detection of other between-group differences or predictive relationships between group assignment or demographic variables and outcomes of interest was limited by the small sample size. It was also not feasible to compare pregnant women and those parenting infants within groups at pretest and posttest periods to determine whether there were statistically significant differences owing to the small sample size. However, our approach helped to accomplish our goals for the pilot study and has facilitated a robust planning process for scaling to the full randomized controlled trial.

### Conclusions

This pilot study showed that the prototype of the Rosie app is a feasible and usable innovation during pregnancy and postpartum period. This study provides valuable insight into using chatbots to help pregnant women and new mothers of color access reliable information the moment it is requested. Promising pilot results suggest that chatbots may reduce adverse health outcomes among ethnic and racial minoritized mothers; however, additional evaluation is warranted including a planned randomized clinical trial to evaluate the effects of Rosie on maternal and infant outcomes. If successful, chatbots such as Rosie can help address the existing health disparities in maternal and child health that have important intergenerational and downstream health consequences for the nation.

## Appendix

Multimedia Appendix 1 CONSORT (Consolidated Standards of Reporting Trials) checklist.

## Abbreviations

CDC: Centers for Disease Control and Prevention
FAQ: frequently asked question
PAQ: probably asked question
PHQ-9: Patient Health Questionnaire–9
QA: question-answering

## Data Availability

The deidentified versions of data sets generated and analyzed during this study are available from the corresponding author upon reasonable request.
